# Validation of the Quick Mild Cognitive Impairment screen in a primary care clinic sample

**DOI:** 10.1002/alz70857_107244

**Published:** 2025-12-24

**Authors:** Nusha Kheradbin, Liliana Guadagno, Scott Selinger, Heather E Cuevas, Claire Harrison, Joshua T Chang, Robin C. Hilsabeck

**Affiliations:** ^1^ UT Austin Dell Medical School, Austin, TX, USA; ^2^ UT Austin School of Nursing, Austin, TX, USA; ^3^ Dell Medical School at The University of Texas at Austin, Austin, TX, USA; ^4^ UT Health San Antonio, San Antonio, TX, USA

## Abstract

**Background:**

Brief cognitive screening tests (< 5 minutes) are recommended for use in primary care settings due to time constraints. However, most brief measures have been criticized for lack of sensitivity to detect mild cognitive impairment (MCI). The purpose of this study was to explore the validity of the Quick Mild Cognitive Impairment (Qmci) screen, a relatively new brief cognitive screening measure, to accurately identify individuals exhibiting MCI in a primary care sample.

**Method:**

Participants were 107 older adults (average age = 69.3; 69% female; 94% white) without a dementia diagnosis who completed the Qmci during a routine primary care visit. They also completed a brief neuropsychological test battery (NTB) usually later the same day or within 2‐3 weeks. The Qmci has six subtests: Orientation, Word Registration, Clock Drawing, Delayed Word Recall, Verbal Fluency, and Logical Memory. The NTB consisted of six tests assessing language, memory, and executive functioning (EF). Participants with two or more demographically adjusted T scores < 40 within the language, memory, or EF domains or one demographically adjusted T score < 40 in each of the three domains were considered cognitively impaired (CI) on the NTB. Independent samples t‐tests and chi square analyses were used to examine group differences. A receiver operating curve analysis was conducted to determine the classification accuracy of the Qmci.

**Result:**

On the NTB, 76 participants were cognitively normal (CN) and 31 were CI. There were no significant differences in age, education, race, or ethnicity between CN and CI groups. The CN group obtained significantly higher (better) Qmci total scores and Delayed Recall, Verbal Fluency, and Logical Memory scores. The Qmci achieved an area under the curve (AUC) of .75, which was statistically significant. The optimal cutoff score using Youden's Index was < 72, which had a sensitivity of 87% and a specificity of 57%. The recommended cutoff score of < 67 based on memory clinic samples had a sensitivity of 93% and a specificity of 30%.

**Conclusion:**

The Qmci showed adequate classification accuracy for detecting MCI in older adults in a primary care clinic setting.

